# Temporal transcriptome change of *Oncomelania hupensis* revealed by *Schistosoma japonicum* invasion

**DOI:** 10.1186/s13578-020-00420-4

**Published:** 2020-04-17

**Authors:** Xinyu Feng, Lingqian Zhu, Zhiqiang Qin, Xiaojin Mo, Yuwan Hao, Ying Jiang, Wei Hu, Shizhu Li

**Affiliations:** 1 National Institute of Parasitic Diseases, Chinese Center for Disease Control and Prevention, Key Laboratory of Parasite and Vector Biology, National Health and Family Planning Commission, WHO Collaborating Center for Tropical Diseases, National Center for International Research on Tropical Diseases, Shanghai, 200025 People’s Republic of China; 2 Joint Research Laboratory of Genetics and Ecology on Parasites-hosts Interaction, National Institute of Parasitic Diseases-Fudan University, Shanghai, 200025 People’s Republic of China; 3 State Key Laboratory of Genetic Engineering, Ministry of Education Key Laboratory of Contemporary Anthropology, Collaborative Innovation Center for Genetics and Development, School of Life Sciences, Fudan University, Shanghai, 200438 People’s Republic of China

**Keywords:** *Oncomelania hupensis*, *Schistosoma japonicum*, Invasion, Transcriptome

## Abstract

**Background:**

The freshwater snail *Oncomelania hupensis* is the obligate intermediate host for *Schistosoma japonicum* in China. Transcriptomic examination of snail–schistosome interactions can provide valuable information of host response at physiological and immune levels.

**Methods:**

To investigate *S. japonicum*-induced changes in *O. hupensis* gene expression, we utilized high-throughput sequencing to identify transcripts that were differentially expressed between infected snails and their uninfected controls at two key time-point, Day 7 and Day 30 after challenge. Time-series transcriptomic profiles were analyzed using R package DESeq 2, followed by GO, KEGG and (weighted gene correlation network analysis) WGCNA analysis to elucidate and identify important molecular mechanism, and subsequently understand host–parasite relationship. The identified unigenes was verified by bioinformatics and real-time PCR. Possible adaptation molecular mechanisms of *O. hupensis* to *S. japonicum* challenge were proposed.

**Results:**

Transcriptomic analyses of *O. hupensis* by *S. japonicum* invasion yielded billion reads including 92,144 annotated transcripts. Over 5000 differentially expressed genes (DEGs) were identified by pairwise comparisons of infected libraries from two time points to uninfected libraries in *O. hupensis*. In total, 6032 gene ontology terms and 149 KEGG pathways were enriched. After the snails were infected with *S. japonicum* on Day 7 and Day 30, DEGs were shown to be involved in many key processes associated with biological regulation and innate immunity pathways. Gene expression patterns differed after exposure to *S. japonicum*. Using WGCNA, 16 modules were identified. Module-trait analysis identified that a module involved in RNA binding, ribosome, translation, mRNA processing, and structural constituent of ribosome were strongly associated with S. japonicum invasion. Many of the genes from enriched KEGG pathways were involved in lysosome, spliceosome and ribosome, indicating that *S. japonicum* invasion may activate the regulation of ribosomes and immune response to infection in *O. hupensis*.

**Conclusions:**

Our analysis provided a temporally dynamic gene expression pattern of *O. hupensis* by *S. japonicum* invasion. The identification of gene candidates serves as a foundation for future investigations of *S. japonicum* infection. Additionally, major DEGs expression patterns and putative key regulatory pathways would provide useful information to construct gene regulatory networks between host-parasite crosstalk.

## Introduction

Schistosomiasis is a zoonotic parasitic disease estimated to affect over 250 million people worldwide [1]. It is transmitted by snails infected with worms of the trematode genus Schistosoma. *Oncomelania hupensis* (Gastropoda: Rissooidea), mainly distributed in China’s Yangtze river basin and its south, is the only intermediate host of *Schistosoma japonicum*, which plays a key role in the transmission of schistosomiasis in China [2, 3]. The number of Schistosomiasis patients and the burden of the disease have dramatically reduced since the establishment of People’s Republic of China (P.R.C), together with the national schistosomiasis control strategy shift from the morbidity control strategy to an integrated strategy in 2004 [4–6]. However, the disease remains endemic in some regions and contributes to a major public health concern in China. Moreover, there are still *O. hupensis* snails distributed in the ecologically complicated environments, such as lakeshore, marshland and mountainous areas. The breeding and spread of *O. hupensis* is the hotbed for the transmission and retransmission of Schistosomiasis, even after elimination.

The life cycle of schistosomiasis is complicated. Its life cycle necessitates the presence of an intermediate host-a fresh water mollusk [7]. After released from the egg, the miracidium, the first larval stage in the life cycle, begin to search for its specific intermediate host. It penetrates the snail, subsequently multiply asexually into multicellular sporocysts and later into cercarial larvae [8, 9]. The *S. japonicum* miracidium develop into sporocysts larva in the *O. hupensis* within a week. The glands, ganglia, eye spots and apical papilla get degenerated in this stage, except the ciliated epidermis replaced by neodermis (tegument). After about 30 days infected snails release free swimming cercariae in response to sunlight. These can penetrate the skin of the mammalian host within 12–24 h and finally invade the definite host [10, 11]. In addition to the parasite itself, it is well-known that the infection in *O. hupensis* depends on many factors, such as parasite-snail compatibility, environmental aspects, individual snail defense capacity and specificity, and so on. Thus, the schistosome-snail interaction is a key area of research of biomedical significance.

With the help of tremendous breakthroughs in molecular research, the application of next-generation sequencing (NGS) technology in genomics, transcriptomics, epigenomics and metagenomics have been performed to drive forward our understanding of many processes and potential regulatory mechanisms in schistosomiasis involved both the parasite and host. The genome of *Biomphalaria glabrata* published in May 2017 [12], which has dramatically improved the efficiency of gene discovery in snail. In a recent study Wang et al. tried to determine modification of neuropeptides in the snail *B. glabrata* ganglia nervous system before and after infection with *Schistosoma mansoni* miracidia [13]. They found that the neuropeptides and precursor proteins, particularly those involved in snail reproduction were heavily down regulated and less abundant in prepatent snails compared to non-infected snails. For *O. hupensis,* transcriptomic studies by RNA-seq have been performed after molluscicide treatment. Many genes involved in key processes associated with biological regulation and innate immunity have been identified, which would benefit for elucidating the molluscicidal mechanism [14]. Another study by comparing the differences in gene expression between the hilly and marshland snails revealed that there is a potential relationship between expression profiling and ecological adaptation of the snail that may have implications for schistosomiasis control in future [15]. Our team reported preliminary analysis of raw data of *O. hupensis* before and after *S. japonicum* invasion, and 178,436,865 clean reads with a Q20 percentage of 87.90% were obtained [16]. However, we failed to conduct in-depth analysis due to sequence contamination of *S. japonicum*. Nevertheless, the key molecular mechanisms involved in the parasite–snail interaction, the subsequent larva development, and the main molecular events occurring during infection establishment in the intermediate host have not been investigated so far.

Here, we provide and further analyze global view of the transcriptomes at two key time point to determine which physiological changes and immune responses occur in the snail after infections with *S. japonicum* miracidia. Understanding the pathogen–host interactions and critical molecular events on these time nodes during infection establishment in prepatent snails will provide new insights for the development of novel markers of detecting infected snails and anti-schistosome strategies.

## Methods

### Sample collection and preparation

**Fig. 1:**
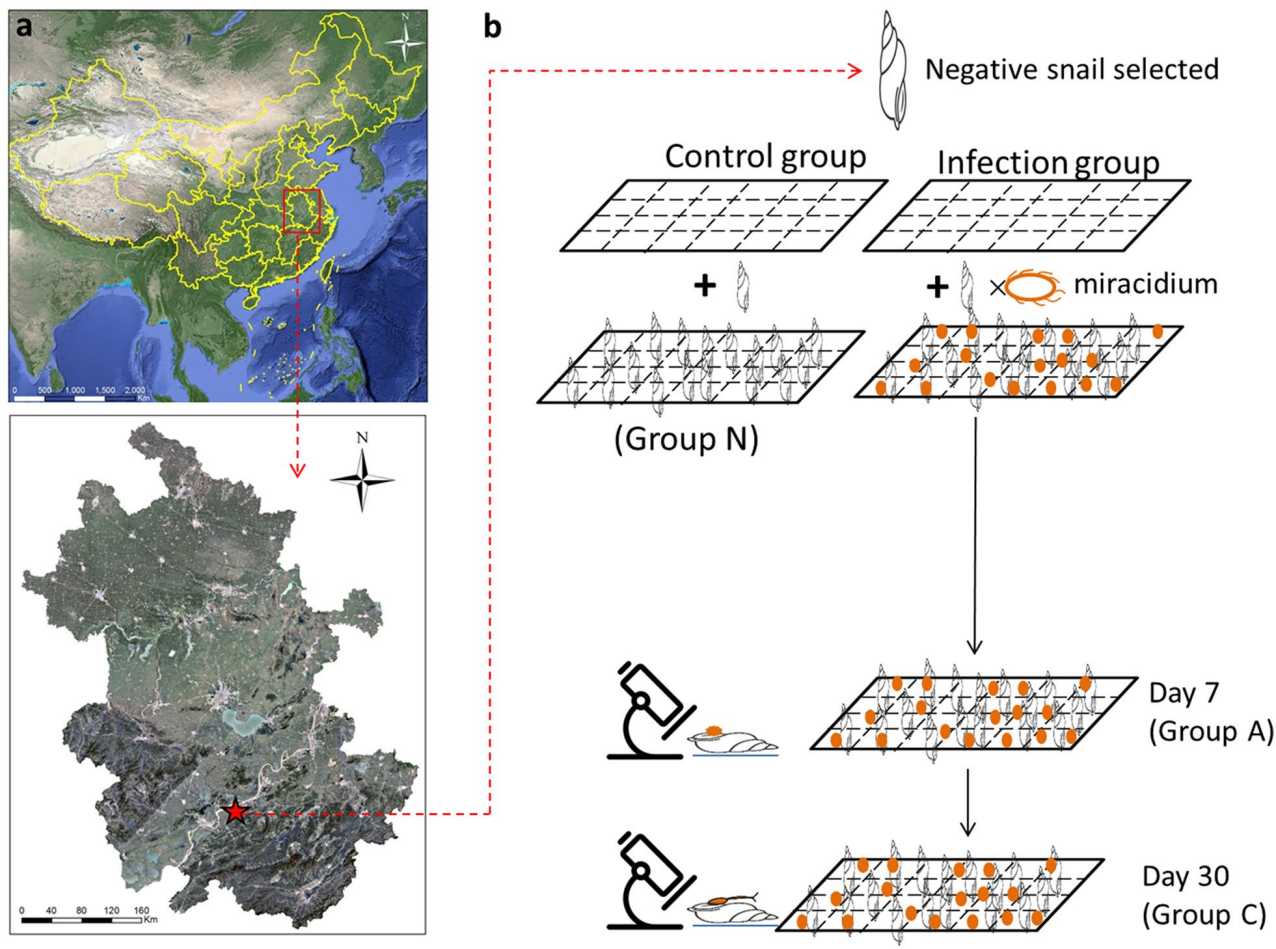
**a** Geographical location of the sampling site in Guichi district in Anhui province; **b** overall workflow for collection of non-infected and infected *O. hupensis.* Control group is Group N(naïve stage), while infection group include Group A (initial infection stage, 7 dpi) and Group C (late infection stage, 30 dpi)

*Oncomelania hupensis* specimens were originally collected from Guichi district, Chizhou city, Anhui province during April 2008 to February 2012 (Fig. 1a). The collected samples were reared in the insectary for a week, and confirmed by cercaria shedding test. After excluding a natural infection, all snails were used for subsequent investigations. The freshly hatched miracidia were harvested from the liver of New Zealand white rabbits as describe in [17], then pooled and placed in a container with de-chlorinated water. Subsequently, snails were separately placed in a 24-well culture plate of de-chlorinated water, and then added 10 miracidia to each well, resulting in a snail to miracidia ratio of ~1:10. Snails were illuminated under light for 6 –8 h at 25 − 30 °C conditions. Snails were then removed and placed into 20 × 30 cm trays and cultured. De-chlorinated water was added daily to the culture trays to keep the moisture at a relative humidity of 85%. Throughout the experiment, snails were checked daily and dead ones were removed with forceps and counted. Finally, 10 snails were collected at days 7 and 30 post-infection. Infection was assessed by compression of tissues between two glass plates after removing of shell and examined under microscope to confirm the presence of cercariae or sporocysts (Fig. 1b). The snail soft tissues were pooled (at least three replicates), and then immediately flash frozen and stored for further analysis.

### Transcriptome preprocessing and submission

Briefly, total RNAs were extracted separately from three different groups, designated as N (N1–N3, naïve stage), A (A1–A3, initial infection stage, 7 dpi) and C (C1–C3, late infection stage, 30 dpi), respectively, using TRIzol reagent (Invitrogen, USA) according to the manufacturer’s protocol. Genomic DNA was removed by treating RNA samples with DNase I (Fermentas) prior to cDNA synthesis. RNA quantification and quality were measured by the Nanodrop ND-1000 spectrophotometer (Nanodrop Technologies, Wilmington, DE), combining with Agilent 2100 Bioanalyzer and denaturing gel electrophoresis.

Total RNA (3 µg) from each group was prepared according to the Illumina protocol as described previously. In brief, Magnetic beads with oligo-dT were used to combine the poly-A of the mRNA for purifying the mRNA from the total RNA. Subsequently, poly (A) mRNA was chopped into short fragments using divalent cations under elevated temperature. The fragments were used to synthesize first-strand cDNA with random primers, and first-strand cDNA was transformed into double-strand cDNA by using RNase H and DNA polymerase I according to the manufacturer’s instructions. Short fragments of desirable lengths (200–300 bp) were purified using the QIAquick PCR Extraction Kit (Qiagen, Valencia, CA, USA), which was also used for continued end repair and ‘A’ base addition. The end-repaired DNA fragments were ligated with sequencing adapters through A and T complementary base pairing. AMPure XP beads (Beckman Coulter, Shanghai, China) were used to remove unsuitable fragments. The multiplexed cDNA libraries were checked using PicoGreen (Quantifluor™-STfluorometerE6090, Promega, CA, USA) and fluorospectrophotometry (Quant-iT59PicoGreen dsDNA Assay Kit; Invitrogen, P7589) and quantified with Agilent 2100 (Agilent 2100 Bioanalyzer, Agilent, 2100; Agilent High Sensitivity DNA Kit, Agilent, 5067–4626). The sequencing library was gradually diluted and quantified to 4–5 pM and sequenced using the Illumina NextSeq™ 500 platform at Shanghai Personal Biotechnology Co., Ltd. The transcriptome datasets are available from the NCBI Sequence Read Archive (SRA) under accession number SRR9598637–SRR9598645.

### De novo transcriptome assembly and functional annotation

Clean reads were obtained from raw data by removing adaptor sequences, low quality reads (*Q* < 20) and reads containing adapter or poly-N with the help of cutadapt [18] and fqtrim [19]. De novo assembly of clean data was assembled based on clean reads using Trinity [20] to generate transcripts. For further analysis, the assembled sequences were searched against the NCBI non-redundant nucleotide database (Nt), non-redundant protein database (Nr) and Swiss-Prot database with an E-value < 10^−5^. For contigs annotated as the same references, we removed the shorter fragmented transcripts and retained the longest contigs after removing the redundancy using RSeQC [21]. In order to exclude the contamination of *S. japonica’s* genome, we used Hisat by mapping clean reads onto reference genome and genes for data filtering operations. The obtained contigs were defined as unigenes. Principal component analysis (PCA) was conducted based on the final set of clean datasets obtained from samples representing 3 biologic replicates. The expression of each gene was calculated according to the reads per kilo bases per million reads (RPKM) normalized by log2 transformation and subsequently analysed by the DESeq 2 package. The expression fold change was calculated in Empirical analysis of digital gene expression data in R (edgeR) and used to identify the unigenes that were differentially expressed between paired samples. Genes with expression changes greater than twofold between two groups were considered DEGs (*P*-*adj *< 0.05). GO (gene ontology) annotation of unigenes was performed by the Blast2GO program23 [22]. The metabolic pathways of the unigenes were predicted by Kyoto Encyclopedia of Genes and Genomes (KEGG) [23].

### Weighted gene coexpression network analysis (WGCNA)

WGNCA approach was applied in this study to find gene co-expression modules based on the normalized gene expression values for each sample. Samples from each group were collected to identify modules that had different expression patterns, where each module represents a group of genes having similar co-expression patterns. Next, the genes from the modules with coefficients higher than 0.5 were selected and subjected to GO and KEGG enrichment analysis.

### Quantification of mRNA expression level

Quantification of nine genes was randomly selected and performed by means of qRT-PCR as described before (Additional file 1: Table S1). Total RNA samples for quantification of gene expression were the same as the samples prepared for cDNA library construction. Briefly, the total RNA was extracted using TRIzol Reagent (Invitrogen, USA). 1 μg of total RNA was reverse-transcribed TaKaRa Super RT Kit. All reactions were assayed in three biological and technical replications and performed in an ABI-2700 (Applied Biosystems, USA) using SYBR Green qPCR kit (Invitrogen, USA), according to the manufacturer’s instructions. Forward (F) and reverse (R) of specific primers used to amplify genes of interest in the qRT-PCR reactions are listed in Additional file 1: Table S1. The first step of amplification was denaturation at 95 °C for 10 min, followed by 40 cycles with 15 s at 95 °C and 10 s at 60 °C. Melting curve analysis was carried out using following conditions: 1 min at 95 °C, 65 °C for 2 min and progressive increase from 65 °C to 95 °C to ensure that a single product was amplified in each reaction. The relative expression level of mRNA was normalized to the internal control of *O. hupensis* 18S and calculated by using the 2^−ΔΔCt^ method. Finally, the results were statistically analyzed using One-way ANOVA and then Post Hoc Multiple Comparisons in SPSS 19.0.

## Results

### Overview of sequencing data

**Fig. 2:**
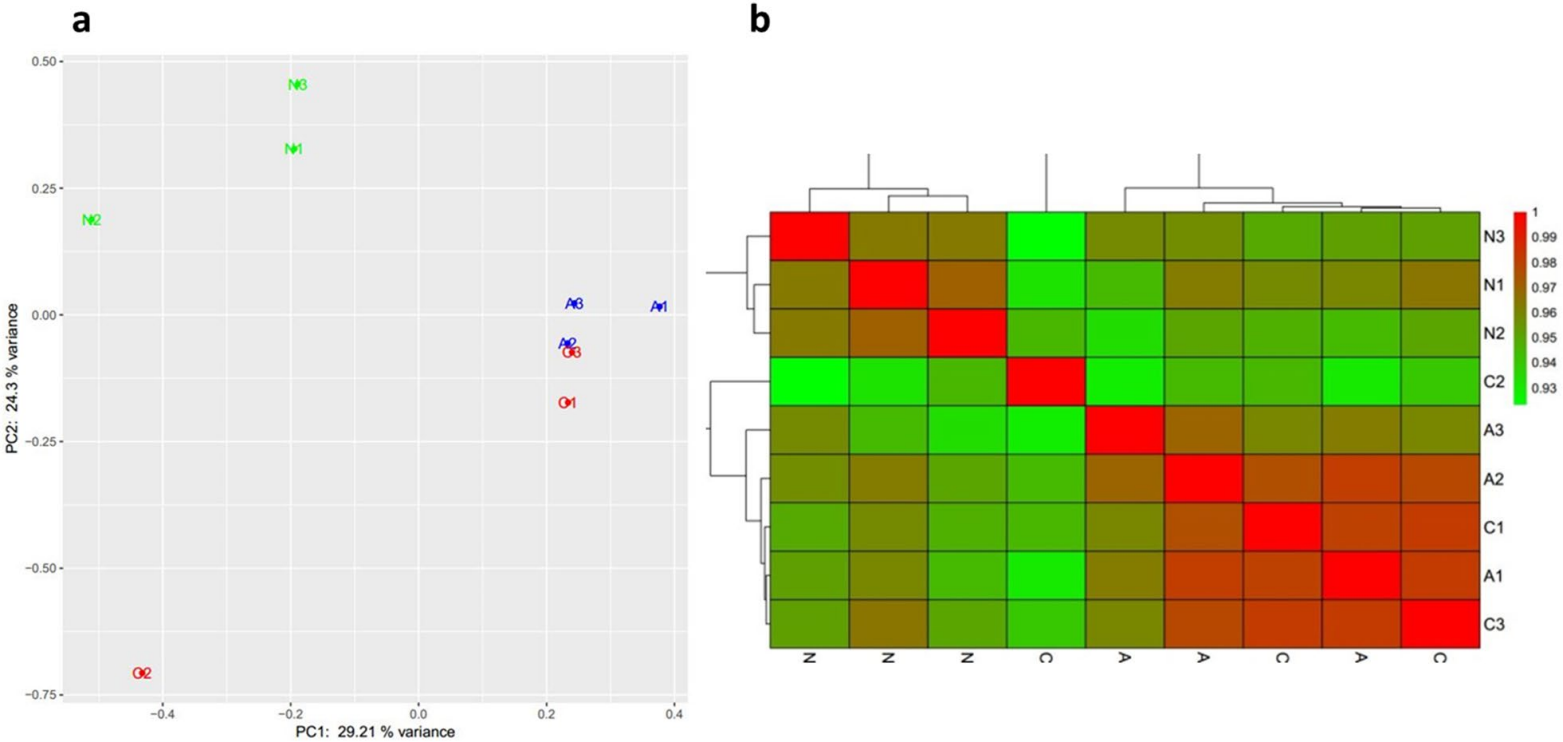
Correlating transcriptomes from three group samples. Group N (N1-N3, naïve stage), Group A (A1–A3, initial infection stage, 7 dpi) and Group C (C1–C3, late infection stage, 30 dpi) (**a**) Principal Components Analysis (PCA) on the whole set samples; (**b**) pearson correlation coefficient heat map on each sample

To achieve a comprehensive view of gene expression dynamics of *O. hupensis* by *S. japonicum* invasion, we incorporated 9 tissue/carcass samples at two key time point and one control sample. After trimming out contamination of *S. japonicum* (by mapping clean reads to *S. japonicum*’s genome using Hisat), low-quality reads and repeats, 56,226 validated transcripts (transcripts could be detected in at least 5 samples) were screened from total 92,144 transcripts. The size distribution of the reads was similar between each two libraries. PCA was also performed on the sequencing dataset, which clear segregated the control sample and *S. japonicum* challenged samples (Fig. 2a). Notably, PCA is unable to discriminate one sample of later of infection stage. PCA showed one sample of later of infection stage (C2) was linearly separated. We then computed the Pearson correlation coefficients between the paired samples, and plotted them as the heat maps as shown in Fig. 2b, where the most poorly correlated (median pairwise *r*^2^ = 0.92) pair was between C2 and N3.

### Temporal transcriptome dynamics before and after infection

**Fig. 3:**
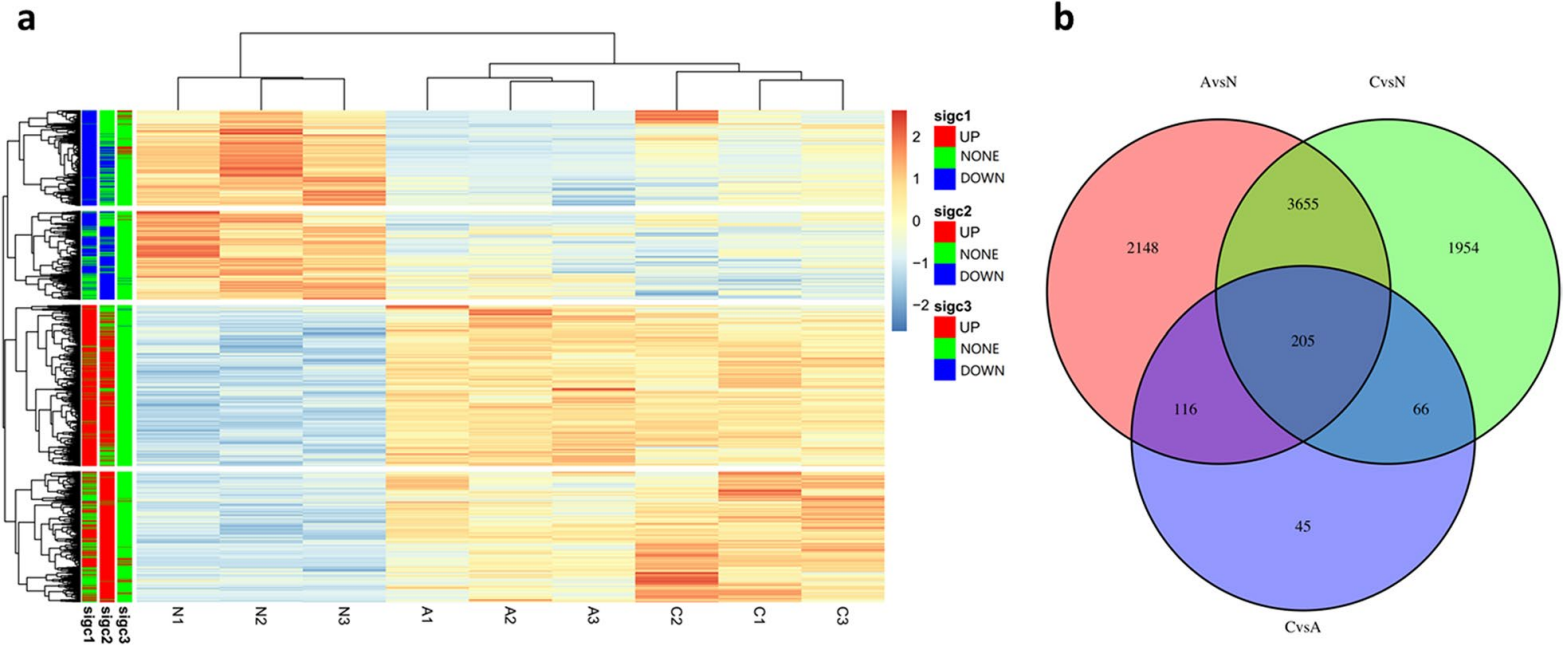
**a** Hierarchical clustering of the significantly different unigenes revealed distinct patterns of gene expression specific to each of the three groups; **b** a venn diagram showed the number of overlap between the differentially expressed unigenes in pairwise groups. A stands for Group A, N stands for Group N and C stands for Group C

A total of 8189 unigenes were found to be differentially expressed. To investigate gene expression pattern change and identify the critical genes in the *O. hupensis* response to *S. japonicum* invasion, pairwise comparisons were performed between each time point (A vs N, C vs A and C vs N). Among these unigenes, 2466 were downregulated and 3658 were upregulated by |log2FoldChange| > 1 (*P *< 0.05) in group A compared with that in group N. 1732 unigenes were downregulated and 4148 were upregulated in group C compared with that in group N. Whereas, only 38 unigenes were downregulated and 394 were upregulated in group C compared with that in group A. The fold-change ranged from − 11.64 to 12.95 for the downregulated and upregulated unigenes (Additional file 2: Table S2). Hierarchical clustering of the significantly different unigenes (two-way ANOVA, FDR-adjusted *P*-adj value < 0.01) reveals distinct patterns of gene expression specific to each of the three groups (Fig. 3a). We also found that 205 unigenes overlapped among differentially expressed unigenes of paired groups (Fig. 3b).

### Function analysis of DEGs

**Fig. 4:**
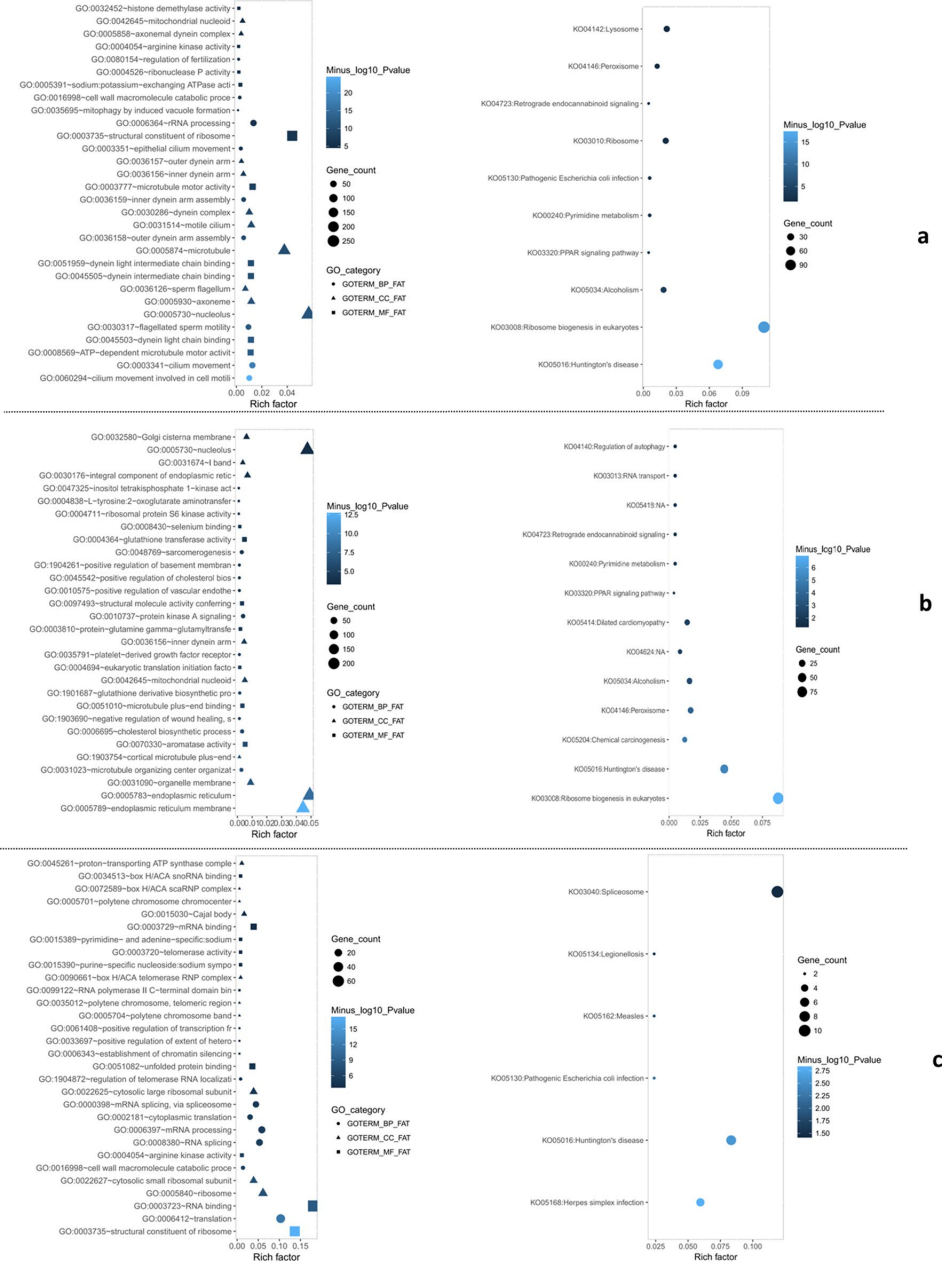
Gene ontology and KEGG enrichment analysis of DEGs. **a** Gene ontology and KEGG enrichment analysis of GEGs from group A compared to group N; **b** gene ontology and KEGG enrichment analysis of GEGs from group C compared to group N; **c** Gene ontology and KEGG enrichment analysis of GEGs from group C compared to group A

Firstly, the annotated genes were searched in the eggNOG database to determine their functional classification macroscopically [24]. To further explore the functions of the DEGs, we performed a Gene Ontology (GO) and KEGG(Kyoto Encyclopedia of Genes and Genomes)enrichment analysis [23, 25]. When group A compared with that in group N, nucleolus, axoneme and sperm flagellum were the most represented subcategories in cellular components; cilium movement involved in cell motility, cilium movement and flagellated sperm motility were the most represented in biological processes; ATP-dependent microtubule motor activity, dynein intermediate chain binding and dynein light chain binding were most represented in molecular function. Subsequently, KEGG pathway enrichment analysis for the DEGs revealed that ribosome biogenesis in eukaryotes, lysosome and ribosome was the top three enriched categories (Fig. 4a). When group C compared with that in group N, endoplasmic reticulum membrane, nucleolus and endoplasmic reticulum were the most represented subcategories in cellular components; microtubule organizing center organization, cholesterol biosynthetic process and negative regulation of wound healing were the most represented in biological processes; aromatase activity, microtubule plus-end binding and eukaryotic translation initiation factor 2 alpha kinase activity were most represented in molecular function. KEGG pathway enrichment analysis for the DEGs revealed that ribosome biogenesis in eukaryotes, peroxisome and chemical carcinogenesis were the top three enriched categories (Fig. 4b). For group C vs group A, in general, only a small part of unigenes dysregulated significantly, which probably reflects the strong reduction in overall transcription. Ribosome, cytosolic small ribosomal subunit and cytosolic large ribosomal subunit were the most represented subcategories in cellular components; cell wall macromolecule catabolic process, RNA splicing and mRNA processing were the most represented in biological processes; structural constituent of ribosome, RNA binding and arginine kinase activity were most represented in molecular function. KEGG pathway enrichment analysis for the DEGs revealed that spliceosome was the most enriched category (Fig. 4c).

### The key gene network associated with infection identified by WGCNA

**Fig. 5:**
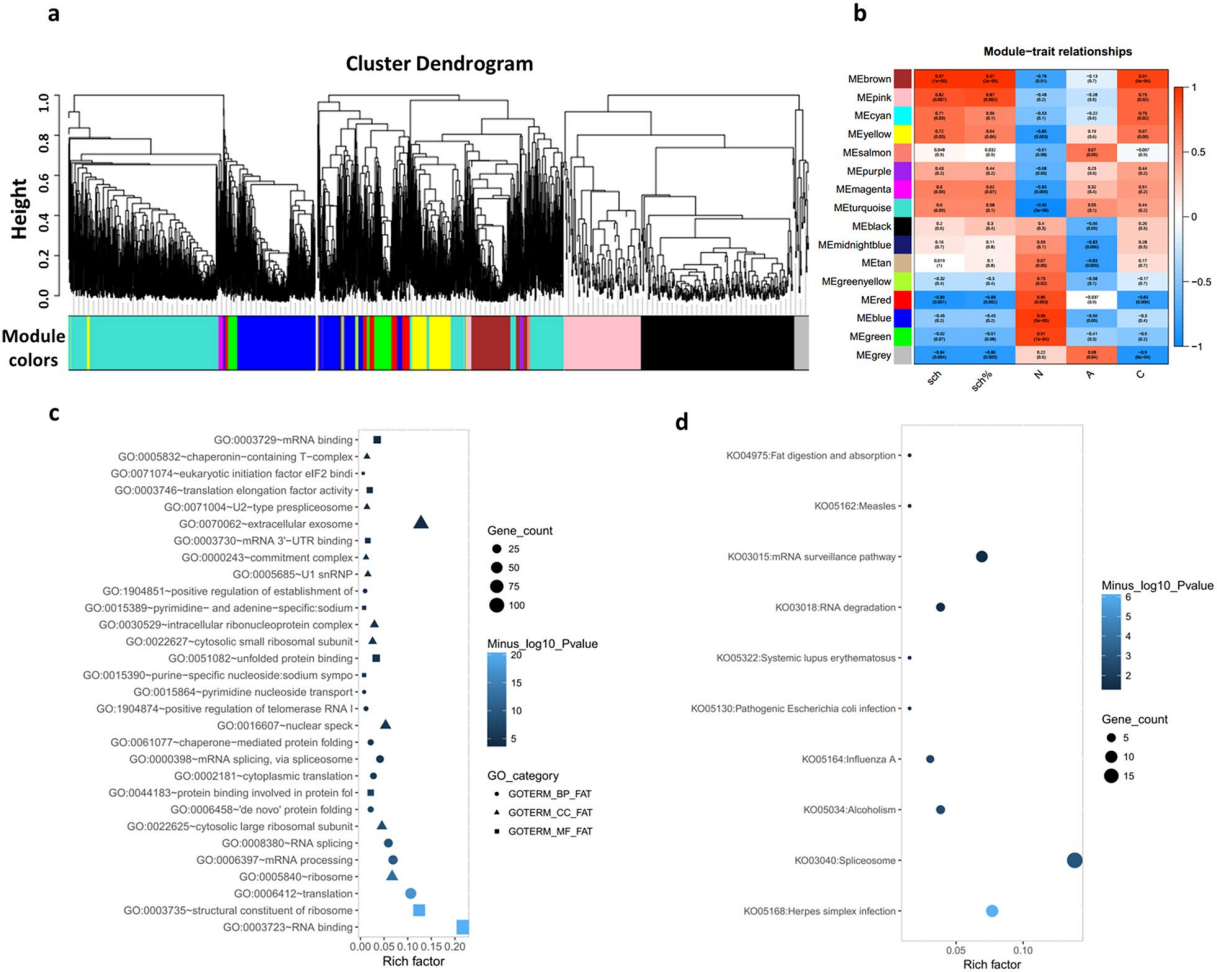
WGCNA of differentially expressed genes from transcriptome response of *O. hupensis* by *s. japonicum* invasion. **a** Dendrogram of modules identified by WGCNA; **b** Correlation matrix of module eigengene values and phenotypes; (**c**) and (**d**) gene ontology and KEGG enrichment analysis of GEGs from the brown module

In our analysis, to identify the gene network that was highly associated with *S. japonicum* invasion, we detected 16 modules by WGCNA based on the RNA-Seq data (Fig. 5a). Based on the results above, the green, blue, red, black, yellow and tan modules significant correlated with group N phenotype, and the grey, salmon and turquoise, together with the brown, pink, cyan, and yellow had significant relationship with group C and group A phenotype, respectively (Fig. 5b). These results indicated that genes that had high correlation with the brown module were strongly associated with *S. japonicum* invasion. The genes from the modules with coefficients higher than 0.5 were selected and subjected to GO and KEGG enrichment analysis. We found that GO function terms were enriched in RNA binding, ribosome, translation, mRNA processing, structural constituent of ribosome (Fig. 5c), and spliceosome, mRNA surveillance pathway, RNA degradation and pathogenic infection were enriched in the KEGG analysis (Fig. 5d), indicating that *S. japonicum* invasion may activate the regulation of ribosomes and response to infection-related mechanisms in *O. hupensis*.

### Quatification of DEGs

**Fig. 6:**
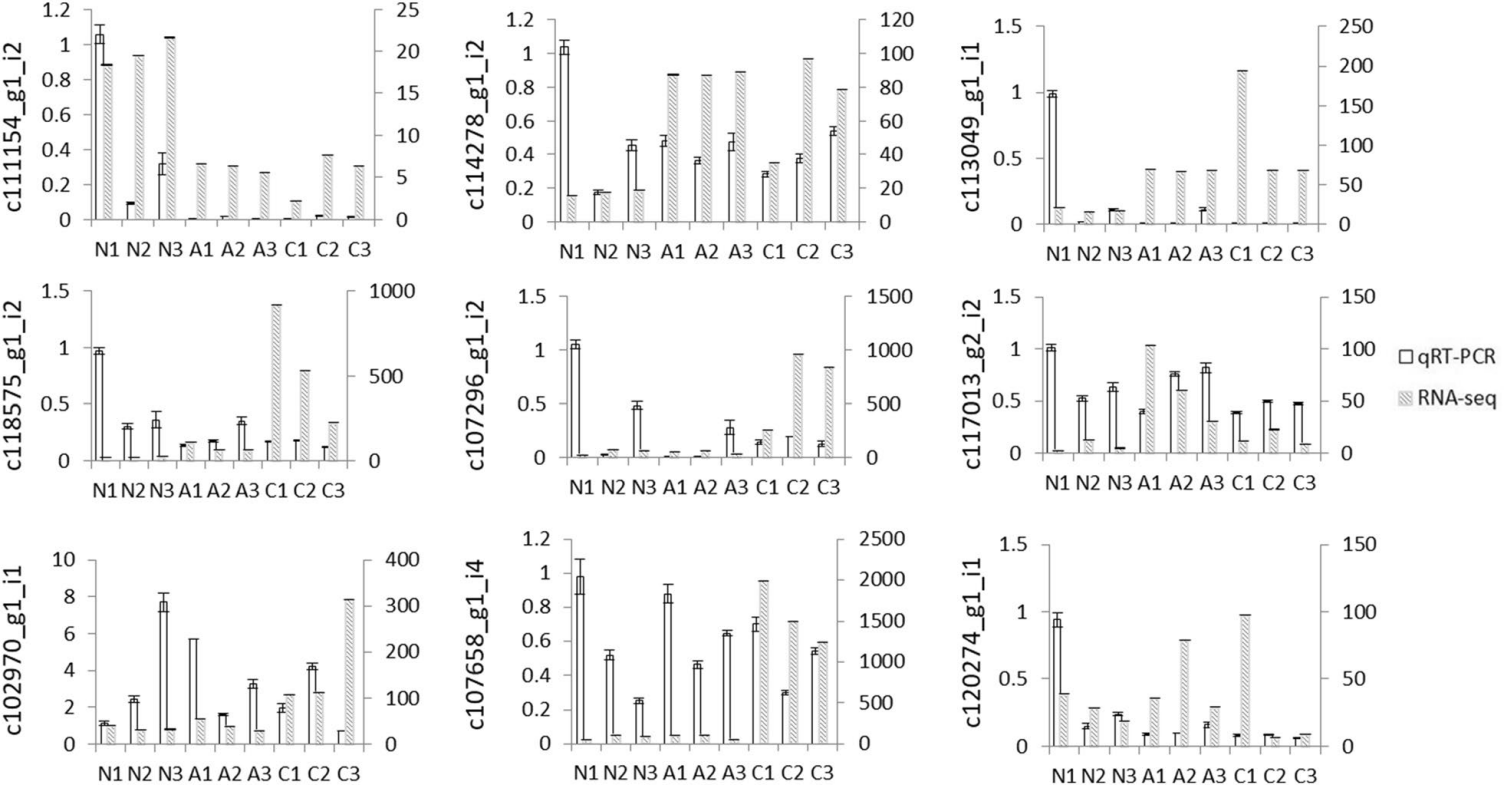
Validation of gene expression profiles by qRT-PCR and RNA-seq in three different groups. The transcript levels of unigenes were calculated relative to the amount of 18 small nuclear after normalization. The real time PCR data with bars represent the mean ± SD from three independent experiments. The left y-axis represents the relative gene expression level by qRT-PCR, and the right y-axis indicates relative gene expression level of RNA-seq

To validate the reliability of the transcriptome data and expression level of the identified unigenes, nine unigenes with significantly differential expression were selected for qRT-PCR as described previously. The result (Fig. 6) showed that most of samples showed similar expression patterns as those revealed by our sequencing result. Data are presented as the mean ± standard deviation of three replicates. The unigene c111154_g1_i2 annotated as “tripartite motif-containing protein 59-like” has the highest expression in group N. While c114278_g1_i2 annotated as “protein transport protein Sec61 subunit gamma” and c113049_g1_i1 annotated as “ATP-binding cassette sub-family F member 2-like” has relatively high expression in group A and C, respectively. All of the selected genes were successfully amplified. However, the expression levels of some samples (for example, N1 in group N) by qRT-PCR were inconsistent with that of sequencing results, which might be caused by individual variation, amplification efficiency and other unconfirmed factors.

## Discussion

The neglected tropical disease of schistosomiasis remains one of the most intractable public health concerns, which exert serious impact on global disease burden. *S. japonicum* has a complex life-cycle involving two hosts. *Oncomelania hupensis* is of great medical significance as it is the unique intermediate host for the transmission of schistosomiasis in China. The invasion of *O. hupensis* by *S. japonicum* can lead to massive physiological and immune changes. In this study, we provide a comprehensive view of transcriptomic changes by *S. japonicum* invasion. We are interested to find what molecular components were modified upon infection, as well as to know the corresponding related defense and immune mechanisms in parasite-host interactions.

The larval stages of *S. japonicum* generally develop within its first intermediate host–*O. hupensis.* Thus, the invasion of trematode may have a negative effect on snails due to mechanical cell damage and competition on nutrient access. The 7 days and 30 days are the two key time points for the success of *S. japonicum* development and infection in the *O. hupensis*. Generally, the *S. japonicum* miracidium need a week to develop into sporocysts larva within the *O. hupensis* accompanied by many organs differentiate and deform. Additionally, free-swimming cercariae are released from infected snails after about 30 days after two rounds of asexual reproduction. So these temporal transcriptome changes may reflect the difference in the schistosome-snail interaction in the initial and later infection stages. In this study, we have found that the transcriptome profiles of infected group (A and C) were distinctively different from naïve group N. Among the three studied groups, group A and C showed distinct different gene expression patterns when compared to group N; whereas in group C vs group A, the difference of significant changed pattern was not distinct with great difference. One possible explanation is that the interplay between host-parasite intensified during the migration of the larval stages of trematodes inside *O. hupensis* at the later stages. We observed that PCA showed one sample of later of infection stage (C2) was linearly separated with two others. We conclude this may be caused by the relatively small biological repeat of samples in the study. To validate our sequencing results, we performed qRT-PCR analysis using the RNA sample used for RNA-seq. Although some unigenes showed similar expression patterns as those revealed by our sequencing analysis, however, relative expression level of unigenes for N1 from the group N by qRT-PCR were inconsistent with the sequencing results. We assumed that this might be caused by the experimental conditions, or analytical error in both small RNA NGS and the qRT-PCR, but some other unknown reasons cannot be excluded.

The unavailability of the genome of *O. hupensis* hinders investigation on the regulation of gene expression and regulation. Particularly, some aspects of the biology of *O. hupensis* as well as its relationship to the parasite *S. japonicum* interaction are still poorly explored. Thus, this study will greatly assist in better understanding of the host-parasite interaction. In our study, the upregulated and downregulated genes of *O. hupensis* upon *S. japonicum* infection provide a global view of the regulatory interactions. Henceforth, comparison of two key time point transcriptomes with uninfected stage was performed. We found that thousands of genes were differentially expressed, and more genes were upregulated following *S. japonicum* challenge. These findings are in line with other previous studies on *Biomphalaria glabrata,* which is the major intermediate snail host for *S. mansoni* [26]. For example, 41 open reading frame expressed sequence tags (ORESTES) libraries from different tissue types that had either remained unexposed or had been exposed to *S. mansoni* revealed that the number of sequences obtained from infected group is more than that of uninfected group [27]. Anne E Lockyer et al. [28] identified 98 differentially expressed genes or gene clusters using snail haemocyte RNA of 2 to 24 h post-exposure to *S. mansoni* hybridized to the custom made cDNA microarray. Also, differential expression of putative Argonaute, Drosha, Piwi, Exportin-5 and Tudor genes at different snail developmental stages and during infection with *S. mansoni* were observed. The authors found that relative expression of some homologous genes were significantly upregulated in *S. mansoni* compared to 5 days group [15]. By contrast, only a few study focused on the transcriptomic studies in the snail *O. hupensis*, for instance, Zhao et al. have characterized the transcriptome profiling of *O. hupensis* from the two distinct habitats and identified thousands of genes that show either increased or decreased expression [15]. A RNA-Seq experiment revealed that gene expression of *O. hupensis* showed significantly effects exposed to two kinds of molluscicides [14]. Together these data demonstrate the highly dynamic nature of transcriptomic profile in response to environment, infection and molluscicide. Overall RNA-Seq analysis results characterized 8189 differentially expressed unigenes. Among them, 2466 and 1732 downregulated unigenes, and 3658 and 4148 upregulated unigenes were found to be differentially expressed in group A and C respectively when compared with group N. Some DEGs showed significant expression fold-change value of > 10, and enriched in important GO function and KEGG pathways terms, such as RNA binding, ribosome, translation, mRNA processing, and lysosome, spliceosome and ribosome. However, these DEGs should be further investigated as potential markers associated with detection of *S. japonicum* in *O. hupensis*.

The long-standing and complicated host-parasite interaction necessities the needs to study the molecular and cellular mechanisms involved in the snail physiological and immune response against schistosome infection. In recent years, a number of conserved signaling pathways and immune-related effectors have been identified in molluscs, especially in *Biomphalaria glabrata*, but few have been reported in *O. hupensis*. Guo et al. reported the functional properties of hemocyanin of *O. hupensis*, and showed that OhH exhibited o-diphenoloxidase activity after limited proteolysis, and shared carbohydrate epitopes with glycoconjugates of *S. japonicum* [29]. Then, in 2012, Zhang et al. identified three goose-type (g-type) lysozymes from expressed sequence tags (ESTs) of a gastropod *O. hupensis* [30]. Thioredoxin peroxidase (*TPx*) gene of *O. hupensis* was cloned and expressed, and the recombinant TPx protein shows a certain antioxidant activity [31]. Interestingly, in our study, we also found a homologue of thioredoxin domain-containing protein (unigene75336, participating in protein processing in endoplasmic reticulum related pathway) was significantly up-regulated in group A and group C with high fold change (above 5). MIF, a homologue of mammalian macrophage migration inhibitory factor, which is a constitutively expressed pleiotropic regulator in the host’s antimicrobial defense system and stress response, have successfully identified and functionally characterized from *O. hupensis* (*OhMIF*). Huang et al. found that found that *OhMIF* displays significantly increased expression in snails following challenge with *S. japonicum*. Furthermore, Knockdown of *OhMIF* significantly reduced the percentage of phagocytic cell populations in circulating hemocytes [32]. Lately, Zhao et al. [33] identified 16 Toll-like receptors (*TLRs*) in *O. hupensis*. Of note, after *S. japonicum* challenge, the expression levels of all of the *OhTLRs* were significantly up-regulated at 6 h post-challenge, while they were inhibited and fluctuated at later time points in haemocytes and in other tissues. Additionally; they further determined that Three OhMyD88 genes were highly expressed and up-regulated in haemocytes at the early time-point after *S. japonicum* challenge. Another study found that both the expression of *OhTRP14* and *ROS* level increased significantly in snails following challenge with *S. japonicum* [34], which play a crucial role in the regulation of signaling pathways of NF-κB, mitogen-activated protein kinases (MAPKs), and apoptosis triggered by TNF-α. Similarly, we observed that a homologue of Toll-like receptor 2 (unigene86937, participating in Toll-like receptor signaling pathway) was significantly up-regulated both in group A and group C when compared with Group N. Using GO and KEGG databases, many unigenes were mapped to key processes associated with biological regulation and innate immunity pathways, such as Toll and Imd signaling pathway, PPAR signaling pathway, Ubiquitin mediated proteolysis pathway, and Ribosome biogenesis in eukaryotes.

The number and fold change of genes demonstrates the complex and dynamic transcriptome regulation in *O. hupensis*. However, further investigation into the varied expression of genes in different tissues at different time-point after *S. japonicum* challenge, combing with the critical signal pathways involved, will improve our understanding of gene function and regulation in physical response and innate immunity of *O. hupensis*.

## Conclusion

To investigate the possible defense mechanism of *O. hupensis* by *S. japonicum* invasion, we utilized high-throughput sequencing to identify transcripts that were differentially expressed between infected snails and their uninfected controls. Both at the early and late stages of infection, we obtained many DEGs, which were then subject to GO, KEGG and WGCNA analysis. We identified some important immune signal and physiological processes pathways that likely responded to *S. japonicum* invasion at pre-patent stage. Taken together, results from this study will provide a valuable addition to for future investigations of infection-induced immune response and physiological variation in the *O. hupensis* and snail–schistosome interactions at a substantial level.

## Supplementary information

**Additional file 1: Table S1.** Unigene-specific primers used in qRT-PCR validation.

**Additional file 2**: **Table S2.** Temporal transcriptome dynamics between three different groups.

## Data Availability

The raw data used for assembly are deposited into the National Center for Biotechnology Information (NCBI) Sequence Reads Archive (SRA) under Accession Number SRR9598637-SRR9598645, associated with BioProject Number PRJNA551328.

## References

[1] Wang J, Yu Y, Shen H, Qing T, Zheng Y, Li Q, Mo X, Wang S, Li N, Chai R, Xu B, Liu M, Brindley PJ, McManus DP, Feng Z, Shi L, Hu W (2017). Dynamic transcriptomes identify biogenic amines and insect-like hormonal regulation for mediating reproduction in *Schistosoma japonicum*. Nat Commun..

[2] Yang Y, Zheng SB, Yang Y, Cheng WT, Pan X, Dai QQ, Chen Y, Zhu L, Jiang QW, Zhou YB (2018). The three gorges dam: does the flooding time determine the distribution of schistosome-transmitting snails in the middle and lower reaches of the Yangtze River, China?. Int J Environ Res Public Health..

[3] Wang X, Wang W, Wang P (2017). Long-term effectiveness of the integrated schistosomiasis control strategy with emphasis on infectious source control in China: a 10-year evaluation from 2005 to 2014. Parasitol Res.

[4] Sun LP, Wang W, Zuo YP, Hong QB, Du GL, Ma YC, Wang J, Yang GJ, Zhu DJ, Liang YS (2017). A multidisciplinary, integrated approach for the elimination of schistosomiasis: a longitudinal study in a historically hyper-endemic region in the lower reaches of the Yangtze River, China from 2005 to 2014. Infect Dis Poverty..

[5] Qian C, Zhang Y, Zhang X, Yuan C, Gao Z, Yuan H, Zhong J (2018). Effectiveness of the new integrated strategy to control the transmission of *Schistosoma japonicum* in China: a systematic review and meta-analysis. Parasite..

[6] Zhu G, Fan J, Peterson AT (2017). *Schistosoma japonicum* transmission risk maps at present and under climate change in mainland China. PLoS Negl Trop Dis..

[7] Gryseels B, Polman K, Clerinx J, Kestens L (2006). Human schistosomiasis. Lancet..

[8] Chontananarth T, Wongsawad C (2013). Epidemiology of cercarial stage of trematodes in freshwater snails from Chiang Mai province, Thailand. Asian Pac J Trop Biomed..

[9] Zahoor Z, Lockyer AE, Davies AJ, Kirk RS, Emery AM, Rollinson D, Jones CS, Noble LR, Walker AJ (2014). Differences in the gene expression profiles of haemocytes from schistosome-susceptible and -resistant biomphalaria glabrata exposed to *Schistosoma mansoni* excretory-secretory products. PLoS ONE.

[10] Gray DJ, Ross AG, Li YS, McManus DP (2011). Diagnosis and management of schistosomiasis. BMJ (Clinical Research ed)..

[11] Mao C (1990). Biology of schistosome and control of schistosomiasis.

[12] Tennessen JA, Bollmann SR, Blouin MS (2017). A targeted capture linkage map anchors the genome of the schistosomiasis vector snail, *Biomphalaria glabrata*. G3 (Bethesda, Md)..

[13] Wang T, Zhao M, Liang D, Bose U, Kaur S, McManus DP, Cummins SF (2017). Changes in the neuropeptide content of *Biomphalaria ganglia* nervous system following Schistosoma infection. Parasit Vectors..

[14] Zhao QP, Xiong T, Xu XJ, Jiang MS, Dong HF (2015). De Novo transcriptome analysis of *Oncomelania hupensis* after molluscicide treatment by next-generation sequencing: implications for biology and future snail interventions. PLoS ONE.

[15] Zhao JS, Wang AY, Zhao HB, Chen YH (2017). Transcriptome sequencing and differential gene expression analysis of the schistosome-transmitting snail *Oncomelania hupensis* inhabiting hilly and marshland regions. Sci Rep..

[16] Zhi-Qiang Q, Xiao-Jing M, Wei G, Shi-Zhu L (2017). Study on transcriptome of *Oncomelania hupensis* before and after *Schistosoma japonicum* invasion I De novo assembly of data by RNA-Seq. Chin J Schisto Control.

[17] Zhao DY, Xu R, Lin JJ, Lu K, Hong Y, Li H, Liu YC, Liu YP, Zhu CG (2014). Distribution characteristics of deposited eggs and pathological changes in viscera of New Zealand white rabbits infected with *Schistosoma japonicum* at different time. Zhongguo xue xi chong bing fang zhi za zhi. Chin J Schisto Control.

[18] Martin MJEJ (2011). Cutadapt removes adapter sequences from high-throughput sequencing reads. EMBnet..

[19] https://ccb.jhu.edu/software/fqtrim/.

[20] Grabherr MG, Haas BJ, Yassour M, Levin JZ, Thompson DA, Amit I, Adiconis X, Fan L, Raychowdhury R, Zeng Q, Chen Z, Mauceli E, Hacohen N, Gnirke A, Rhind N, di Palma F, Birren BW, Nusbaum C, Lindblad-Toh K, Friedman N, Regev A (2011). Full-length transcriptome assembly from RNA-Seq data without a reference genome. Nat Biotechnol.

[21] https://code.google.com/archive/p/rseqc/issues/17.

[22] Conesa A, Gotz S, Garcia-Gomez JM, Terol J, Talon M, Robles M (2005). Blast2GO: a universal tool for annotation, visualization and analysis in functional genomics research. Bioinformatics (Oxford, England)..

[23] Kanehisa M, Goto S (2000). KEGG: kyoto encyclopedia of genes and genomes. Nucleic Acids Res.

[24] Powell S, Szklarczyk D, Trachana K, Roth A, Kuhn M, Muller J, Arnold R, Rattei T, Letunic I, Doerks T, Jensen LJ, von Mering C, Bork P (2012). eggNOG v3.0: orthologous groups covering 1133 organisms at 41 different taxonomic ranges. Nucleic Acids Res..

[25] Harris MA, Clark J, Ireland A, Lomax J, Ashburner M, Foulger R, Eilbeck K, Lewis S, Marshall B, Mungall C, Richter J, Rubin GM, Blake JA, Bult C, Dolan M, Drabkin H, Eppig JT, Hill DP, Ni L, Ringwald M, Balakrishnan R, Cherry JM, Christie KR, Costanzo MC, Dwight SS, Engel S, Fisk DG, Hirschman JE, Hong EL, Nash RS, Sethuraman A, Theesfeld CL, Botstein D, Dolinski K, Feierbach B, Berardini T, Mundodi S, Rhee SY, Apweiler R, Barrell D, Camon E, Dimmer E, Lee V, Chisholm R, Gaudet P, Kibbe W, Kishore R, Schwarz EM, Sternberg P, Gwinn M, Hannick L, Wortman J, Berriman M, Wood V, de la Cruz N, Tonellato P, Jaiswal P, Seigfried T, White R (2004). The gene ontology (GO) database and informatics resource. Nucleic Acids Res..

[26] Queiroz FR, Silva LM, Jeremias WJ, Baba EH, Caldeira RL, Coelho PMZ, Gomes MS (2017). Differential expression of small RNA pathway genes associated with the *Biomphalaria glabrata*/*Schistosoma mansoni* interaction. PLoS ONE.

[27] Lockyer AE, Spinks JN, Walker AJ, Kane RA, Noble LR, Rollinson D, Dias-Neto E, Jones CS (2007). *Biomphalaria glabrata* transcriptome: identification of cell-signalling, transcriptional control and immune-related genes from open reading frame expressed sequence tags (ORESTES). Dev Comp Immunol.

[28] Lockyer AE, Spinks J, Kane RA, Hoffmann KF, Fitzpatrick JM, Rollinson D, Noble LR, Jones CS (2008). *Biomphalaria glabrata* transcriptome: cDNA microarray profiling identifies resistant- and susceptible-specific gene expression in haemocytes from snail strains exposed to *Schistosoma mansoni*. BMC Genomics..

[29] Guo D, Zhang Y, Zeng D, Wang H, Li X, Li Y, Fan X (2009). Functional properties of hemocyanin from *Oncomelania hupensis*, the intermediate host of *Schistosoma japonicum*. Exp Parasitol.

[30] Zhang SH, Zhu DD, Chang MX, Zhao QP, Jiao R, Huang B, Fu JP, Liu ZX, Nie P (2012). Three goose-type lysozymes in the gastropod *Oncomelania hupensis*: cDNA sequences and lytic activity of recombinant proteins. Dev Comp Immunol.

[31] Ma XL, Liu Q, Zhang Y (2012). [Cloning, expression and activity analysis of full-length gene encoding thioredoxin peroxidase from *Oncomelania hupensis*] Zhongguo ji sheng chong xue yu ji sheng chong bing za zhi. Chin J Parasitol Parasit Dis..

[32] Huang S, Cao Y, Lu M, Peng W, Lin J, Tang C, Tang L (2017). Identification and functional characterization of *Oncomelania hupensis* macrophage migration inhibitory factor involved in the snail host innate immune response to the parasite *Schistosoma japonicum*. Int J Parasitol.

[33] Zhao QP, Gao Q, Zhang Y, Li YW, Huang WL, Tang CL, Dong HF (2018). Identification of Toll-like receptor family members in *Oncomelania hupensis* and their role in defense against *Schistosoma japonicum*. Acta Trop.

[34] Cao Y, Huang S, Peng W, Lu M, Peng W, Lin J, Tang C, Tang L (2018). Identification and functional characterization of thioredoxin-related protein of 14 kDa in *Oncomelania hupensis*, the intermediate host of *Schistosoma japonicum*. Mol Biochem Parasitol.

