# Supplementary material for: Temporal transcriptome change of Oncomelania hupensis revealed by Schistosoma japonicum invasion
Source: Cell Biosci. 2020 Apr 17;10:58. doi: 10.1186/s13578-020-00420-4 (PMC7165382; doi:10.1186/s13578-020-00420-4)
Supplement: Supplementary file 1 — Additional file 1: Table S1. Unigene-specific primers used in qRT-PCR validation. [file 13578_2020_420_MOESM1_ESM.docx]

T**able S1**. Unigene-specific primers used in qRT-PCR validation

| **Primer** | **Sequence** |
| --- | --- |
| c111154_g1_i2-F | CACCAAACAACGCACCTCAG |
| c111154_g1_i2-R | GCTTGTTCCACGTCAGAGGA |
| c114278_g1_i2-F | TTGGCTAGTGAACTCGGCTG |
| c114278_g1_i2-R | CACGGCAGTAGGGATTCTCC |
| c113049_g1_i1-F | ATCGCTCTGTGACTGGTGTG |
| c113049_g1_i1-R | GGCCATCTCTCGACGCAATA |
| c118575_g1_i2-F | ATGCTACTCCAAGGCACCAC |
| c118575_g1_i2-R | ACAGGACTCTGGGTTGGTCT |
| c107296_g1_i2-F | GAATGTCAATGTCCGCCGTC |
| c107296_g1_i2-R | CAGCGAACGGGTGTTATCCA |
| c117013_g2_i2-F | AGTCCCAAACGCTCACTAGC |
| c117013_g2_i2-R | TTCAGGCACAAGGTGGTCAA |
| c102970_g1_i1-F | TCGCTAGAGCAGACGCTATC |
| c102970_g1_i1-R | GGAGGGAAAATCCTCAGCGT |
| c107658_g1_i4-F | GCCAGCAGGTGTTTACAACG |
| c107658_g1_i4-R | CGTCTGCGGTATCTTGACGA |
| c120274_g1_i1-F | GCAATAGCTGCATGAGTGGC |
| c120274_g1_i1-R | CTCCGTAAGAAAGCGTTGCG |
| 18S-F | CGTCCTTTTGGTGACTCTGG |
| 18S-R | TGGATGTGGTAGCCGTTTCTC |
